# Cerebrospinal fluid haptoglobin levels and outcome after aneurysmal subarachnoid haemorrhage: Evidence from Mendelian randomization

**DOI:** 10.1371/journal.pone.0329287

**Published:** 2025-08-05

**Authors:** Gbenga A. Kayode, Loukas Zagkos, Godspower Oboli, Sonya Abraham, Gregory Kato, Quazi Ataher, Evangelia Farmakioti, Lothar Tremmel, Stephen Burgess

**Affiliations:** 1 CSL BEHRING L.L.C., King of Prussia, Pennsylvania, United States of America; 2 Lane Clark & Peacock LLP, London, United Kingdom; 3 CSL Innovation GmBH, Marburg, Germany; 4 MRC Biostatistics Unit, School of Clinical Medicine, University of Cambridge, Cambridge, United Kingdom; Universitatsklinikum Regensburg, GERMANY

## Abstract

**Background:**

Subarachnoid haemorrhage (SAH) poses a life-threatening risk, contributing to half of all haemorrhagic strokes, with aneurysmal SAH (aSAH) affecting approximately 6 individuals per 100,000 annually. Following aSAH, the influx of plasma haptoglobin into the cerebrospinal fluid (CSF) is insufficient to sequester the released free haemoglobin in the subarachnoid space and avoid its neurotoxic effects. Exogenous administration of haptoglobin could be a therapeutic strategy for improving outcomes after aSAH.

**Methods:**

Using individual level data in the UK Biobank and genetic summary statistics from the largest relevant cohorts, Mendelian randomization analysis was conducted to explore the associations of genetically predicted CSF haptoglobin with the risk of catastrophic aSAH (i.e., fatal aSAH or non-fatal aSAH with at least one of: hemiparesis, aphasia, apraxia, or visual field defects within 7 days) and secondary traits, including stroke-related outcomes and imaging markers of brain swelling.

**Results:**

Higher levels of genetically predicted CSF haptoglobin was associated with lower risk of catastrophic aSAH in multi-ancestry (OR: 0.79, 95% CI: 0.65 to 0.96, *p* = 0.019) and White British sample analyses (OR: 0.78, 95% CI: 0.63 to 0.95, *p* = 0.013), while the lack of association with related traits supported effects specific to outcomes after aSAH. A proof-of-concept analysis showing associations of genetically predicted haptoglobin with haemoglobin in plasma validated the proposed mechanism.

**Conclusions:**

Using human genetic data, we provide evidence to support a causal role of higher CSF haptoglobin in improving outcomes after aSAH. However, there was no evidence for an effect on the risk of aSAH or related cerebrovascular events. These findings support haptoglobin as a potential treatment in aSAH to mitigate the neurotoxic effects of free haemoglobin and improve outcomes.

## Introduction

Subarachnoid haemorrhage (SAH) is a life-threatening condition responsible for up to half of all haemorrhagic strokes [1]. Most cases (85%) of non-traumatic SAH result from the spontaneous rupture of saccular aneurysms which are abnormal bulges at areas of weakness in the walls of intracranial blood vessels [2,3]. Aneurysmal SAH (aSAH) is associated with a high mortality rate (30–40%) and with significant disability among survivors [4].

Aneurysmal rupture triggers a cascade of biological events leading to serious clinical complications. The mass effect of blood released directly into the cerebrospinal fluid (CSF) within the subarachnoid space rapidly elevates intracranial pressure [5]. Additionally, the release into the CSF of spasmogenic substances like haemoglobin may lead to both intracranial micro- and macrovascular vasospasm via nitric oxide depletion [6], with cerebral hypoperfusion and resultant delayed cerebral ischaemia (DCI). Finally, released haemoglobin may cause brain injury by inducing microthrombus formation [6], direct neurotoxicity, e.g., via oxidative damage from its haem component and downstream products [7], and cortical spreading depolarisation which results in depression of brain activity [8].

Haptoglobin is a large multimeric protein encoded by the *HP* gene on chromosome 16 functioning as a potent detoxifier of free haemoglobin [9]. Following aSAH, plasma-derived haptoglobin entering the CSF is insufficient relative to free haemoglobin released into the subarachnoid space [6]. Hence, increasing CSF haptoglobin has been proposed as a potential therapeutic intervention in aSAH [10]. Previous studies investigating the potential role of haptoglobin in haemoglobin-mediated brain injury have largely been limited to animal and in vitro studies [6,7,11–15]. Moreover, the only human study with intrathecal haptoglobin intervention conducted to date suffered limitations due to a small sample size and was susceptible to bias owing to its observational design [16].

Mendelian randomization (MR) is an analytical approach that aims to overcome limitations that arise in conventional epidemiological studies, such as bias due to confounding and reverse causation. To achieve this, MR leverages naturally occurring variation in genetic variants to investigate a causal link between an exposure and an outcome, under three core instrumental variables assumptions: (1) genetic variants used as instruments must be robustly associated with the exposure; (2) genetic variants must not be associated with confounding factors of the exposure-outcome relationship and (3) genetic variants should associate with the outcome exclusively through their effect on the exposure. In this work we used the MR paradigm to investigate the association of genetically predicted CSF haptoglobin levels with the risk of catastrophic aSAH, defined as aSAH with fatality or non-fatal aSAH with any of the following neurological complications within 7 days: hemiparesis, aphasia, apraxia, or visual field defects, but also explore associations with other related health outcomes.

## Methods

### Ethics approval and participant consent

Ethical approval and participant consent were obtained in the original GWAS studies. No human participants were recruited for this study. UK Biobank has approval from the North-West Multi-centre Research Ethics Committee (MREC) as a Research Tissue Bank (RTB) approval. This study was conducted under UK Biobank application number 150618. Patients or the public were not involved in the design, or conduct, or reporting, or dissemination plans of our research.

### Study objectives

**Fig 1:**
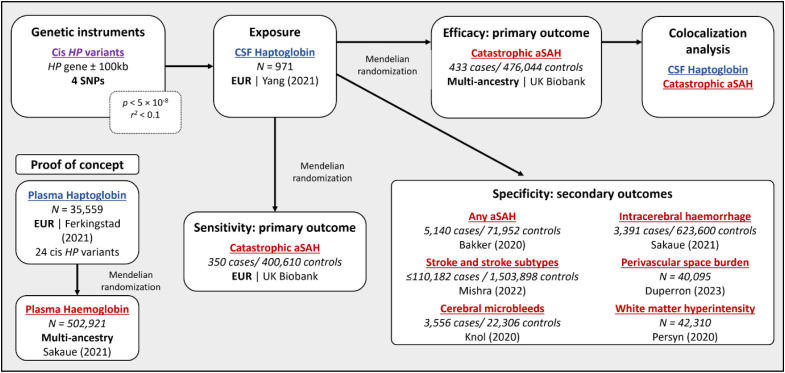
Study design: We investigated the effect of cerebrospinal fluid (CSF) haptoglobin perturbation on the risk of catastrophic aneurysmal subarachnoid haemorrhage (aSAH) using the Mendelian randomization framework in multi-ancestry and White British only populations. As proof of concept, we explored the effect of genetically predicted plasma haptoglobin. To evaluate the specificity of this association, we investigated several related cerebrovascular traits as secondary outcomes. As sensitivity, we conducted colocalization analysis to assess whether CSF haptoglobin and catastrophic aSAH shared a causal variant.

This study used genetic variation at the haptoglobin (*HP*) locus to investigate the causal relationship between CSF haptoglobin and selected outcomes within the MR framework. First, we conducted Mendelian randomization to evaluate the validity of the approach by testing the association of genetically predicted plasma haptoglobin with plasma haemoglobin levels. Then, we investigated the association of genetically predicted CSF haptoglobin with the risk of catastrophic aSAH in multi-ancestry individuals and White British participants only. To explore specificity of associations for outcome after aSAH, we examined associations of genetically predicted CSF haptoglobin with related outcomes, including any aSAH, stroke and stroke subtypes, imaging markers of brain swelling, microbleeds and white matter hyperintensity. The study design is depicted in Fig 1.

### Genetic instrument selection

To instrument CSF haptoglobin, we selected single nucleotide polymorphisms (SNPs) that are associated with CSF haptoglobin abundance, below the genome-wide significant level (*p* < 5 × 10^−8^) and were located within the *HP* gene (genomic position chr16:72,088,491−72,094,954, GRCh37/hg19 by Ensembl) or 100kb before the start or after the end of the gene. Genetic variants were clumped using a pair-wise linkage disequilibrium (LD) r^2^ < 0.1 from the 1000 genomes project phase 3 European LD reference panel [17]. If genetic instruments were missing from the outcome datasets, we used proxy SNPs having LD r^2^ > 0.9 with the missing SNPs. To instrument plasma haptoglobin, we selected SNPs associated with plasma haptoglobin levels using the same selection criteria as above.

### Data sources

Table 1 summarises the data sources utilised in this study. We leveraged summary statistics on CSF haptoglobin relative abundance from a GWAS study in 971 individuals (mean ± standard deviation age of 69.4 ± 9.3 years, 53% women), recruited from the Washington University School of Medicine in St. Louis, United States, including 249 patients with Alzheimer’s disease and 717 cognitively normal controls [18]. We obtained genetic data for plasma haptoglobin measured in 35,559 participants of Icelandic ancestry, using the aptamer-based SomaScan v4 platform [19]. For the primary outcomes, catastrophic and any aSAH, genetic association estimates were produced using individual level data in the UK Biobank (UKB) under application no. 150618. UKB is a prospective cohort study that involved more than 500,000 participants between the ages of 40–69 years [20]. Information on the genotyping process, imputation and data quality control has been described elsewhere [21]. For any aSAH, we also obtained genetic summary statistics from the largest meta-analysis GWAS to date, conducted in 5,140 aSAH cases and 71,952 controls [22]. Genetic association data for intracerebral haemorrhage (cases: 3,391 / controls: 623,600) were obtained from a multi-ancestry GWAS meta-analysis of UK Biobank and FinnGen [23]. Genetic data for plasma haemoglobin were also obtained from the same study, measured in 502,921 participants in UK Biobank and Biobank Japan [23]. Genetic associations with stroke and stroke subtypes, including all-cause stroke (110,182 cases/ 1,503,898 controls), ischaemic stroke (86,668 cases/ 1,503,898 controls), large artery stroke (9,219 cases/ 1,503,898 controls), cardioembolic stroke (12,790 cases/ 1,503,898 controls) and small vessel stroke (13,620 cases/ 1,503,898 controls) were retrieved from a multi-ancestry GWAS in the GIGASTROKE consortium [24]. Last, we obtained multi-ancestry genetic data for imaging markers of brain health, including brain microbleeds (meta-analysis of 14 GWAS in 3,556 cases and 22,306 controls) [25], white matter hyperintensity in 42,310 UKB participants [26] and three perivascular space burden traits in 40,095 individuals from the CHARGE consortium [27]. All GWAS data used in this study were conducted in multi-ancestry populations, unless otherwise stated.

**Table 1:** Data sources utilized in this study.

Trait	N cases / controls	Cohort / Data source
**Exposure**		
CSF haptoglobin	791	Washington University cohort [18]
Plasma haptoglobin	35,599	deCODE [19]
**Primary outcomes**		
Catastrophic aSAH	433 / 476,092	UKB (full cohort)^†^
Catastrophic aSAH	350 / 400,610	UKB (White British subset)^†^
**Secondary and sensitivity outcomes**		
Plasma haemoglobin	502,921	UKB and BBJ [23]
**Haemorrhagic outcomes**		
Any aSAH	1,648 / 476,092	UKB (full cohort)^†^
Any aSAH	5,140 / 71,952	Meta-analysis of 25 cohorts [22]
Intracerebral haemorrhage	3,391 / 623,600	UKB and FinnGen [23]
**Stroke**		
All-cause stroke	110,182 / 1,503,898	GIGASTROKE [24]
Ischaemic stroke	86,668 / 1,503,898	GIGASTROKE [24]
Large-artery stroke	9,219 / 1,503,898	GIGASTROKE [24]
Cardioembolic stroke	12,790 / 1,503,898	GIGASTROKE [24]
Small vessel stroke	13,620 / 1,503,898	GIGASTROKE [24]
**Brain imaging markers**		
Brain microbleeds	3,556 / 22,306	UKB, CHARGE and others [25]
White matter hyperintensity	42,310	UKB [26]
Perivascular space burden	40,095	UKB and CHARGE [27]

† Individual-level data. Abbreviations: CSF: cerebrospinal fluid; aSAH: aneurysmal subarachnoid haemorrhage; UKB: UK Biobank; BBJ: Biobank Japan; CHARGE: Cohorts of Heart and Aging Research in Genomic Epidemiology.

### Catastrophic and any aSAH phenotype generation

To construct binary phenotypes for aSAH and catastrophic aSAH, we used data in the UKB from hospital episode statistics (HES) and death registry (UKB data fields 40001 and 40002) and classified participants as cases based on ICD-9 and ICD-10 codes (UKB data fields 41234 and 41259). Individuals with a hospital admission diagnosis recorded as ICD-9 430 or ICD-10 I60, were classified as aSAH cases. Catastrophic aSAH was defined as fatal aSAH (the codes above recorded as the primary or secondary cause of death) or non-fatal aSAH with at least one of the following diagnoses, defined by ICD-10 codes: hemiparesis (G81), aphasia (R47), apraxia (R48.2), or visual field defects (H53.4), occurring within 7 days after the incident of aSAH. Participants with aSAH occurrence who did not have any complications up to 7 days after aSAH were excluded from the analysis. We also excluded participants with genotype missingness greater than 1.5%, leading to 433 cases/ 476,092 controls for catastrophic aSAH and 1,648 cases/ 476,092 controls for any aSAH with complete data. In analysis of White British ancestry individuals only, there were 350 cases/ 400,610 controls for catastrophic aSAH with complete data.

### Statistical analysis

To calculate genetic estimates for catastrophic and any aSAH we used BOLT-LMM [28], a statistical tool which leverages linear mixed model regression that accounts for population stratification and cryptic relatedness in large samples. We regressed aSAH on the genetic instruments, using sex, age and the first 20 genetic principal components as covariates.

#### Mendelian randomization analysis.

As a proof of concept, we explored the association of genetically predicted plasma haptoglobin with plasma haemoglobin. In main analysis, we first tested the association of genetically predicted CSF haptoglobin with our primary outcome, i.e., catastrophic aSAH. The full (multi-ancestry) instrument-outcome association data were used in the first iteration of this analysis, followed by an MR restricted to White British individuals only. Second, we performed MR analyses testing the association of genetically predicted CSF haptoglobin with any aSAH and secondary neurovascular outcomes. MR estimates were obtained using the random-effects inverse-variance weighted method [29], which aggregates the effect estimates from the four genetic instrumental variants, weighting them by the inverse of their variance. To avoid over-precise MR estimates due to the correlation between the genetic variants used as instruments, we conducted MR using the random-effects inverse-variance weighted method, incorporating a correlation matrix in the analysis. The MR weighted median method was conducted as sensitivity analyses to assess the robustness of the main method findings [30]. To assess potential bias in the MR estimates due to unbalanced horizontal pleiotropy [31], we used the p-value of the MR-Egger intercept [32,33], with p-values below 0.05 indicating the presence of pleiotropy. Furthermore, we explored reported associations of the four genetic variants used to instrument for CSF haptoglobin with potential confounders in the UK Biobank, FinnGen and GWAS catalog. We also calculated Cochran’s Q statistic p-value and the I^2^ statistic, considering ≤0.05 (Q p-value) and ≥0.75 (I^2^) indicators of heterogeneity [34]. A p-value of 0.05 was applied as the threshold for statistical significance for primary outcome analysis. For secondary outcomes, we considered a Bonferroni-corrected p-value of 0.05/12 = 0.004 for statistical significance, describing p-values between 0.004 and 0.05 as “nominally significant”. This accounts for the 12 associations tested, including plasma haemoglobin, 2 haemorrhagic outcomes (any aSAH, intracerebral haemorrhage), 5 stroke outcomes (all-cause, ischaemic, cardioembolic, large artery and small vessel stroke) and 5 brain imaging derived markers (brain microbleeds, white matter hyperintensity, and white matter, hippocampal, and basal ganglia perivascular space burden) (Table 2). All MR effect estimates are reported per one standard deviation (SD) higher genetically predicted haptoglobin. The R package ‘TwoSampleMR’ v.0.5.7 [34] was used to run the analysis.

**Table 2:** Mendelian randomization results for the associations between genetically predicted CSF haptoglobin and binary/continuous endpoints.

			IVW				Pleiotropy & Heterogeneity	
Binary Endpoint	Ancestry	First author	OR	95% CI lower	95% CI upper	p-value	EggerIntP	QP
Catastrophic aSAH	multi	–	0.79	0.65	0.96	0.019	0.389	0.350
Catastrophic aSAH	White British	–	0.78	0.63	0.95	0.013	0.554	0.913
Any aSAH	multi	–	0.95	0.84	1.07	0.400	0.198	0.195
Any aSAH	multi	Bakker	0.99	0.86	1.13	0.862	0.513	0.002
Cerebral microbleeds (any)	multi	Knol	0.99	0.91	1.07	0.731	0.715	0.970
Cardioembolic stroke	multi	Mishra	0.98	0.94	1.03	0.425	0.497	0.634
Small vessel stroke	multi	Mishra	1.04	0.99	1.08	0.097	0.896	0.846
Stroke	multi	Mishra	0.99	0.98	1.01	0.505	0.923	0.415
Large artery stroke	multi	Mishra	0.95	0.90	1.01	0.086	0.640	0.741
Ischaemic stroke	multi	Mishra	1.00	0.98	1.01	0.667	0.972	0.431
Intracerebral haemorrhage	multi	Sakaue	1.02	0.96	1.09	0.496	0.737	0.574
			**IVW**				**Pleiotropy &** **Heterogeneity**	
**Continuous Endpoint**	**Ancestry**	**First author**	**beta**	**95% CI lower**	**95% CI upper**	**p-value**	**EggerIntP**	**QP**
White matter PSB	multi	Duperron	0.000	−0.011	0.011	0.950	0.433	0.260
Hippocampal PSB	multi	Duperron	0.003	−0.007	0.013	0.520	0.221	0.351
Basal ganglia PSB	multi	Duperron	−0.006	−0.018	0.007	0.382	0.190	0.147
White matter hyperintensity	multi	Persyn	0.006	−0.019	0.031	0.651	0.552	0.688
Plasma haemoglobin	multi	Sakaue	−0.021	−0.026	−0.016	4.9e-17	0.279	0.001

IVW: Inverse-variance weighted method, OR: odds ratio, CI: confidence interval, EggerIntP: MR-Egger intercept p-value, QP: Cochran’s Q statistic p-value, aSAH: aneurysmal subarachnoid haemorrhage, PSB: Perivascular space burden.

#### Colocalization analysis.

Colocalization is a statistical approach to assess whether the genetic predictors of two traits overlap (known as colocalization) or are distinct (known as non-colocalization). A finding of colocalization is consistent with a shared mechanism pathway linking the traits, whereas non-colocalization is indicative of either horizontal pleiotropy or linkage disequilibrium with another pathway. In the context of MR, colocalization analysis provides an additional layer of evidence supporting the causal inference drawn by significant MR estimates. The “coloc” method reports several key outputs, including the posterior probability of a causal variant for trait 1 only (PP-H1), the posterior probability of a causal variant for trait 2 only (PP-H2), the posterior probability of non-colocalization (PP-H3), and the posterior probability of colocalization (PP-H4) [35]. High values (close to 1) of PP-H4 indicate colocalization, which is supportive of a causal relationship; high values of PP-H3 indicate non-colocalization, which opposes a causal relationship; high values of PP-H1 or PP-H2 indicate lack of strong evidence supporting or opposing a causal relationship. In the latter case, the quantity PP-H4/(PP-H3 + PP-H4), which represents the posterior probability of colocalization conditional on the presence of a causal variant for both traits, can be calculated to assess whether the evidence favours or opposes colocalization [36]. We conducted colocalization analysis to assess whether CSF haptoglobin colocalized with catastrophic aSAH in the *HP* gene (genomic position chr16:72,088,491−72,094,954, GRCh37/hg19 by Ensembl) or 100kb either way of the gene.

## Results

**Fig 2:**
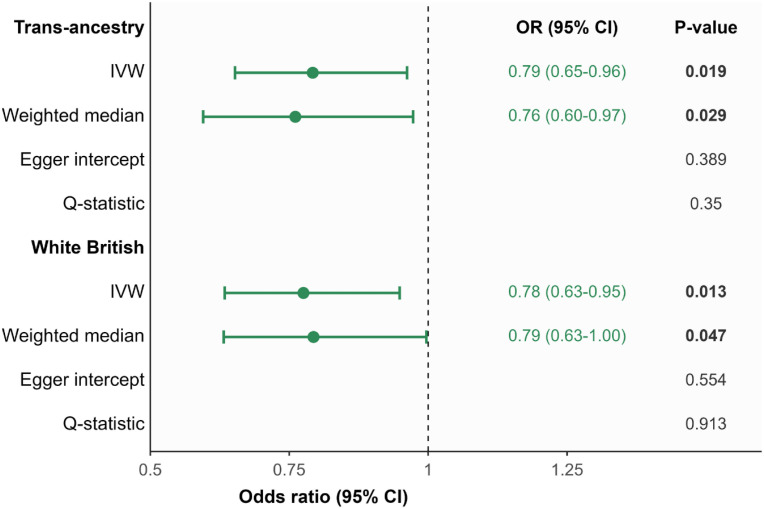
Mendelian randomization estimates for the association of higher genetically predicted cerebrospinal fluid (CSF) haptoglobin with catastrophic aneurysmal subarachnoid haemorrhage (aSAH) in multi-ancestry and White British only UK Biobank participants. Mendelian randomization (MR) estimates are expressed as odds ratio per one standard deviation higher genetically predicted CSF haptoglobin. Higher genetically predicted CSF haptoglobin was associated with lower risk of catastrophic aSAH using inverse variance weighted and weighted median MR methods. There was no evidence of pleiotropy or heterogeneity, as indicated by the non-significant Egger intercept and Q-statistic.

Four SNPs around the *HP* gene region were used as instruments for CSF haptoglobin, with an average F-statistic of 45.9 and 24 SNPs for plasma haptoglobin with an average F-statistic of 919.5 (S1 Table). The mean variance of CSF haptoglobin explained by the four SNPs was 6.3%. S2 Table presents the summary statistics across the four genetic instruments, derived from GWAS conducted using individual-level UK Biobank data for catastrophic and any aSAH. Higher genetically predicted plasma haptoglobin was associated with lower plasma haemoglobin (beta: −0.021, 95% CI: −0.026 to ‑0.016, *p* = 4.9 × 10^-17^), serving as proof of concept and a positive control analysis. Higher genetically predicted CSF haptoglobin was associated with lower risk of catastrophic aSAH in multi-ancestry (odds ratio [OR]: 0.79, 95% CI: 0.65 to 0.96, *p* = 0.019) and White British sample analyses (OR: 0.78, 95% CI: 0.63 to 0.95, *p* = 0.013) (Fig 2). In secondary MR analysis in multi-ancestry participants, the random-effects inverse-variance weighted method incorporating a correlation matrix showed a consistent estimate (OR: 0.79, 95% CI: 0.64 to 0.97, p = 0.026). There was no association between genetically predicted CSF haptoglobin and the risk of any aSAH, using both UKB participant data (OR: 0.95, 95% CI: 0.84 to 1.07, *p* = 0.40) and consortium genetic summary statistics (OR: 0.99, 95% CI: 0.86 to 1.13, *p* = 0.86) for any aSAH. Genetically predicted CSF haptoglobin did not associate (*p* > 0.05) with stroke or stroke subtypes, white matter hyperintensity, cerebral microbleeds, or other perivascular space burden phenotypes, suggesting some degree of outcome selectivity, though not definitively establishing biological specificity with the primary outcome (catastrophic aSAH) (Table 2 and S3 Table). In our analysis there was a statistical power of 68.6% to detect an effect corresponding to an odds ratio of 0.79.

In sensitivity analyses, we observed consistent MR weighted median estimates for catastrophic aSAH in multi-ancestry (OR: 0.76, 95% CI: 0.60 to 0.97, *p* = 0.029) and White British samples (OR: 0.79, 95% CI: 0.63 to 1.00, *p* = 0.047). Cochran’s Q statistic was non-significant (*p* > 0.05) for all significant associations, indicating no heterogeneity within the effect estimates. Additionally, there was no evidence of horizontal pleiotropy as quantified by the MR-Egger intercept (*p* > 0.05) (S3 Table). Exploring reported genetic associations of our instrument for CSF haptoglobin, we found strong associations with haptoglobin measurements, low density lipoprotein cholesterol (LDL-c) levels, apolipoprotein A1, and several haematological traits, including red blood cell count, haemoglobin levels and concentration and haematocrit (S4 Table). However, as genetic variants are located in the *HP* gene region, any pleiotropic associations are likely to represent vertical pleiotropy rather than horizontal pleiotropy (i.e., these associations represent downstream effects of haptoglobin, not competing causal pathways). Hence, we do not believe that these associations bias our analysis.

Colocalization analyses did not provide strong evidence for a causal variant for catastrophic aSAH (PP-H1 = 0.99), as variants in the region of interest did not approach necessary significance levels. However, when only considering the hypotheses of colocalization and non-colocalization, the analysis provided suggestive evidence of colocalization (PP-H4/(PP-H3 + PP-H4)= 0.69) (S5 Table).

## Discussion

This study investigated the hypothesised relationship between genetically predicted CSF haptoglobin levels and the risk of catastrophic aSAH and related phenotypes. We report that higher genetically predicted CSF haptoglobin levels are associated with lower risk of catastrophic aSAH, but not associated with risk of aSAH, intracerebral haemorrhage, stroke, nor with imaging markers of brain swelling, microbleeds, or white matter injury. There was evidence of colocalization conditional on the presence of a causal variant for both CSF haptoglobin and catastrophic aSAH, which further supported our hypothesis of CSF haptoglobin serving as likely causal risk factor for improving outcomes after aSAH.

Epidemiological studies on the association between CSF haptoglobin and aSAH risk, post-aSAH complications or related brain imaging phenotypes are limited. The observations from a Japanese single-arm trial in 27 patients that examined the effect of haptoglobin on post-aSAH vasospasm are in concordance with our findings [16]. Sixteen (59%) of the 27 patients to whom haptoglobin was administered topically to surrounding intracranial arteries during aneurysmal surgery showed subsequent angiographic evidence of improved vasospasm. However, evidence from this study is weakened by its small sample size, and other important limitations, including lack of a comparator group untreated with haptoglobin and potential selection bias as nine of the 16 responders already showed decreasing vasospasm before haptoglobin treatment. Furthermore, although haptoglobin genotype influences CSF haptoglobin levels [37], observational studies have not provided convincing evidence of an association of *HP* genotype with post-aSAH outcomes [10,38]. This may be attributed to the physiologically low levels of CSF haptoglobin which may not be sufficient to substantially impact outcomes [39].

Preclinical studies have predominantly demonstrated protective effects of haptoglobin on post-aSAH outcomes. Studies in mice have demonstrated reduction in haemoglobin-mediated neurotoxicity and small-vessel vasospasm after haptoglobin treatment [7,13,40]. Similarly, a sheep model study suggested that CSF haptoglobin administration halts haemoglobin-induced cerebral vasospasm [6]. Conversely, following haemoglobin exposure, an in vitro study showed a sevenfold increase in neuronal loss with haptoglobin treatment [14]. Notably, these neurons expressed the CD163 receptor responsible for the uptake of haptoglobin-haemoglobin complexes [9], a function primarily performed in the brain by microglia [38]. While CD163 expression in human neurons after intracranial haemorrhage is unclear [14], this has been demonstrated in animal models [41,42]. However, a more recent in vitro study contradicts this, suggesting that haptoglobin prevents, rather than enhances, haemoglobin-mediated toxicity in neuronal cultures [43]. Specifically, it was demonstrated that haptoglobin restored ATP levels and preserved neurite integrity when haemoglobin was sequestered into haptoglobin-haemoglobin complexes. These more recent findings align with the established biological mechanism of haptoglobin sequestering free haemoglobin to prevent its toxic effects. Our genetic evidence supports these findings, suggesting that higher CSF haptoglobin levels may have a protective effect on downstream outcomes, specifically in reducing the risk of fatality or serious neurological complications once aSAH has already occurred.

To evaluate whether the observed association between CSF haptoglobin and outcomes after aSAH reflected a broader cerebrovascular effect, we examined a range of related phenotypes, including stroke subtypes, white matter hyperintensities, cerebral microbleeds, and perivascular space burden. These traits were selected because they are well-characterised imaging or clinical markers of cerebrovascular and small vessel disease to explore potential horizontal pleiotropy. While the lack of association across these outcomes provides some evidence against broad pleiotropic effects across cerebrovascular conditions, it does not conclusively establish biological specificity, and the potential for pleiotropic effects remains.

Our work has several strengths. The utilisation of genetic data in an MR framework enabled powerful statistical analyses, offering insights into the potential administration of haptoglobin into the CSF post-aSAH occurrence to enhance outcomes. In addition, to our knowledge, this is the first study examining the relationship between genetically predicted CSF haptoglobin levels and the risk of catastrophic aSAH and related outcomes in humans. Importantly, unlike conventional observational studies, our use of MR limits the potential of bias due to reverse causality or confounding to affect our estimates.

However, there are important limitations. Mendelian randomization captures the effects of lifelong predisposition to higher CSF haptoglobin levels, rather than the short-term, time-sensitive elevations that would result from acute therapeutic administration. This distinction limits the direct clinical translatability of our findings, as they do not account for the pharmacokinetics, dosing, or timing required for haptoglobin to exert neuroprotective effects in the acute post-aSAH setting. Moreover, we instrumented haptoglobin perturbation using aptamer-based protein quantification which does not distinguish between its three proteoforms (Hp 1–1, 2–1, and 2–2) [44] but instead quantifies the relative abundance of (any) haptoglobin. As the different proteoforms exhibit distinct biological properties, failure to differentiate between these proteoforms may influence the interpretation of our findings. Moreover, the genetic instrument for CSF haptoglobin was derived from a relatively small, European ancestry cohort, which included individuals with Alzheimer's disease. This restricts the generalisability of our findings, particularly across ancestries, and introduces the potential for population stratification bias given that some outcome datasets included multi-ancestry populations. Moreover, due to the lack of individual-level data, we were unable to perform stratified analyses by ancestry or age to assess heterogeneity in effect estimates.

Another notable limitation was our inability to directly assess vasospasm as a distinct outcome given its role in mediating haemoglobin toxicity, which may limit the interpretation of our findings. Vasospasm is not captured in administrative healthcare data used in UK Biobank, and no GWAS summary statistics are currently available for this phenotype. Future studies with detailed clinical data capturing vasospasm, coupled with genetic information, could provide valuable insights into this important intermediate mechanism. Furthermore, in this study we defined “catastrophic aSAH” as events occurring within the first seven days of rupture for methodological reasons. This definition enhances phenotype specificity by focusing on complications with a strong temporal relationship to the index haemorrhage and minimising the inclusion of neurological complications unrelated to aneurysmal rupture. While this definition does not capture all DCI cases, particularly those with late onset, any resulting misclassification is expected to be non-differential, likely biasing results toward the null. Additionally, angiographic vasospasm and DCI currently lack GWAS data suitable for MR analysis. Future MR studies should explore whether the protective association of haptoglobin extends beyond this early period. Finally, there were no available datasets for CSF haptoglobin in independent comparable populations to replicate our findings.

In conclusion, our results leveraging human genetic data provide further support for a potential effect of higher CSF haptoglobin levels on reducing risk of adverse post-aSAH outcomes. The utility of haptoglobin as a therapeutic is supported by the results from this MR study and well-designed clinical trials are required to further investigate the efficacy and safety of haptoglobin treatment in the context of aSAH management.

## Supporting information

S1 Table Genetic instrumental variants used in this study. (XLSX)

S2 Table Catastrophic and any aneurysmal subarachnoid haemorrhage genetic summary statistics for the four instruments, produced using BOLT-LMM. (XLSX)

S3 Table Mendelian randomization results. The effect of CSF haptoglobin on the risk of aneurysmal subarachnoid haemorrhage and related health outcomes were explored.
(XLSX)

S4 Table Associations of genetic variants used as instruments with potential confounders in the UK Biobank, FinnGen and GWAS Catalog, below the genome-wide significance threshold (p = 5e-08). (XLSX)

S5 Table Genetic colocalization analysis findings. (XLSX)

S1 File STROBE-MR checklist of recommended items to address in reports of Mendelian randomization studies. (DOCX)
