# Supplementary material for: Cerebrospinal fluid haptoglobin levels and outcome after aneurysmal subarachnoid haemorrhage: Evidence from Mendelian randomization
Source: PLoS One. 2025 Aug 5;20(8):e0329287. doi: 10.1371/journal.pone.0329287 (PMC12324130; doi:10.1371/journal.pone.0329287)
Supplement: S1 File — (DOCX) [file pone.0329287.s006.docx]

**STROBE-MR checklist of recommended items to address in reports of Mendelian randomization studies**^1^ ^2^

| **Item No.** | **Section** | **Checklist item** | **Page No.** | **Relevant text from manuscript** |
| --- | --- | --- | --- | --- |
| 1 | **TITLE and ABSTRACT** | Indicate Mendelian randomization (MR) as the study’s design in the title and/or the abstract if that is a main purpose of the study | 1 | Cerebrospinal fluid haptoglobin levels and outcome after aneurysmal subarachnoid haemorrhage: evidence from Mendelian randomization |
|  | **INTRODUCTION** |  |  |  |
| 2 | **Background** | Explain the scientific background and rationale for the reported study. What is the exposure? Is a potential causal relationship between exposure and outcome plausible? Justify why MR is a helpful method to address the study question | 3 | Subarachnoid haemorrhage (SAH) is a life-threatening condition responsible for up to half of all haemorrhagic strokes.1 Most cases (85%) of non-traumatic SAH result from the spontaneous rupture of saccular aneurysms which are abnormal bulges at areas of weakness in the walls of intracranial blood vessels.2,3 Aneurysmal SAH (aSAH) is associated with a high mortality rate (30-40%) and with significant disability among survivors.4  Aneurysmal rupture triggers a cascade of biological events leading to serious clinical complications. The mass effect of blood released directly into the cerebrospinal fluid (CSF) within the subarachnoid space rapidly elevates intracranial pressure.5 Additionally, the release into the CSF of spasmogenic substances like haemoglobin may lead to both intracranial micro- and macrovascular vasospasm via nitric oxide depletion,6 with cerebral hypoperfusion and resultant delayed cerebral ischaemia (DCI). Finally, released haemoglobin may cause brain injury by inducing microthrombus formation,6 direct neurotoxicity e.g., via oxidative damage from its haem component and downstream products,7 and cortical spreading depolarisation which results in depression of brain activity.8  Haptoglobin is a large multimeric protein encoded by the HP gene on chromosome 16 functioning as a potent detoxifier of free haemoglobin.9 Following aSAH, plasma-derived haptoglobin entering the CSF is insufficient relative to free haemoglobin released into the subarachnoid space.6 Hence, increasing CSF haptoglobin has been proposed as a potential therapeutic intervention in aSAH.10 Previous studies investigating the potential role of haptoglobin in haemoglobin-mediated brain injury have largely been limited to animal and in vitro studies |
| 3 | **Objectives** | State specific objectives clearly, including pre-specified causal hypotheses (if any). State that MR is a method that, under specific assumptions, intends to estimate causal effects | 3 | Mendelian randomization (MR) is an analytical approach that aims to overcome limitations that arise in conventional epidemiological studies, such as bias due to confounding and reverse causation. To achieve this, MR leverages naturally occurring variation in genetic variants to investigate a causal link between an exposure and an outcome. In this work we used the MR paradigm to investigate the association of genetically predicted CSF haptoglobin levels with the risk of catastrophic aSAH, defined as aSAH with fatality or neurological complications within 7 days, and with other related health outcomes. |
|  | **METHODS** |  |  |  |
| 4 | **Study design and data sources** | Present key elements of the study design early in the article. Consider including a table listing sources of data for all phases of the study. For each data source contributing to the analysis, describe the following: |  |  |
|  | a) | Setting: Describe the study design and the underlying population, if possible. Describe the setting, locations, and relevant dates, including periods of recruitment, exposure, follow-up, and data collection, when available. | 4 | This study used genetic variation at the haptoglobin (HP) locus to investigate the causal relationship between CSF haptoglobin and selected outcomes within the MR framework. First, we investigated the association of genetically predicted CSF haptoglobin with the risk of catastrophic aSAH (defined as aSAH with fatality or neurological complications within 7 days, and with other related health outcomes). To explore specificity of associations for outcome after aSAH, we examined associations of genetically predicted CSF haptoglobin with related outcomes, including any aSAH, stroke and stroke subtypes, imaging markers of brain swelling, microbleeds and white matter hyperintensity. The study design is depicted in Figure 1. |
|  | b) | Participants: Give the eligibility criteria, and the sources and methods of selection of participants. Report the sample size, and whether any power or sample size calculations were carried out prior to the main analysis | 5-6 | Table 1 summarises the data sources utilised in this study. For the primary outcomes, catastrophic and any aSAH, genetic association estimates were produced using individual level data in the UK Biobank (UKB) under application no. 150618. UKB is a prospective cohort study that involved more than 500,000 participants between the ages of 40 to 69 years.18 Information on the genotyping process, imputation and data quality control has been described elsewhere.19 For any aSAH, we also obtained genetic summary statistics from the largest meta-analysis GWAS to date, conducted in 5,140 aSAH cases and 71,952 controls.20 We leveraged summary statistics on CSF haptoglobin relative abundance from a GWAS study in 971 individuals (mean ± standard deviation age of 69.4 ± 9.3 years, 53% women), recruited from the Washington University School of Medicine in St. Louis, United States, including 249 patients with Alzheimer’s disease and 717 cognitively normal controls.21 Genetic association data for intracerebral haemorrhage (cases: 3,391 / controls: 623,600) were obtained from a multi-ancestry GWAS meta-analysis of UK Biobank and FinnGen.22 Genetic associations with stroke and stroke subtypes, including all-case stroke (110,182 cases/ 1,503,898 controls), ischemic stroke (86,668 cases/ 1,503,898 controls), large artery stroke (9,219 cases/ 1,503,898 controls), cardioembolic stroke (12,790 cases/ 1,503,898 controls) and small vessel stroke (13,620 cases/ 1,503,898 controls) were retrieved from a multi-ancestry GWAS in the GIGASTROKE consortium.23 Last, we obtained multi-ancestry genetic data for imaging markers of brain health, including brain microbleeds (meta-analysis of 14 GWAS in 3,556 cases and 22,306 controls),24 white matter hyperintensity in 42,310 UKB participants25 and three perivascular space burden traits in 40,095 individuals from the CHARGE consortium.26 All GWAS data used in this study were conducted in multi-ancestry populations, unless otherwise stated.  Catastrophic and any aSAH phenotype generation  To construct binary phenotypes for aSAH and catastrophic aSAH, we used data in the UKB from hospital episode statistics (HES) and death registry (UKB data fields 40001 and 40002) and classified participants as cases based on ICD-9 and ICD-10 codes (UKB data fields 41234 and 41259). Individuals with a hospital admission diagnosis recorded as ICD-9 430 or ICD-10 I60, were classified as aSAH cases. Catastrophic aSAH was defined as fatal aSAH (the codes above recorded as the primary or secondary cause of death) or non-fatal aSAH with at least one of the following diagnoses, defined by ICD-10 codes: hemiparesis (G81), aphasia (R47), apraxia (R48.2), or visual field defects (H53.4), occurring within 7 days after the incident of aSAH. Participants with aSAH occurrence who did not have any complications up to 7 days after aSAH were excluded from the analysis. We also excluded participants with genotype missingness greater than 1.5%, leading to 433 cases/ 476,092 controls for catastrophic aSAH and 1,648 cases/ 476,092 controls for any aSAH with complete data. In analysis of White British ancestry individuals only, there were 350 cases/ 400,610 controls for catastrophic aSAH with complete data. |
|  | c) | Describe measurement, quality control and selection of genetic variants | 5 | We selected instrumental single nucleotide polymorphisms (SNPs) that are associated with CSF haptoglobin abundance, below the genome-wide significant level (p < 5 × 10-8) and were located within the HP gene (genomic position chr16:72,088,491-72,094,954, GRCh37/hg19 by Ensembl) or 100kb before the start or after the end of the gene. Genetic variants were clumped using a pair-wise linkage disequilibrium (LD) r2 < 0.1 from the 1000 genomes project phase 3 European LD reference panel.17 If genetic instruments were missing from the outcome datasets, we used proxy SNPs having LD r2 > 0.9 with the missing SNPs. |
|  | d) | For each exposure, outcome, and other relevant variables, describe methods of assessment and diagnostic criteria for diseases | 5-6 | Table 1 summarises the data sources utilised in this study. For the primary outcomes, catastrophic and any aSAH, genetic association estimates were produced using individual level data in the UK Biobank (UKB) under application no. 150618. UKB is a prospective cohort study that involved more than 500,000 participants between the ages of 40 to 69 years.18 Information on the genotyping process, imputation and data quality control has been described elsewhere.19 For any aSAH, we also obtained genetic summary statistics from the largest meta-analysis GWAS to date, conducted in 5,140 aSAH cases and 71,952 controls.20 We leveraged summary statistics on CSF haptoglobin relative abundance from a GWAS study in 971 individuals (mean ± standard deviation age of 69.4 ± 9.3 years, 53% women), recruited from the Washington University School of Medicine in St. Louis, United States, including 249 patients with Alzheimer’s disease and 717 cognitively normal controls.21 Genetic association data for intracerebral haemorrhage (cases: 3,391 / controls: 623,600) were obtained from a multi-ancestry GWAS meta-analysis of UK Biobank and FinnGen.22 Genetic associations with stroke and stroke subtypes, including all-case stroke (110,182 cases/ 1,503,898 controls), ischemic stroke (86,668 cases/ 1,503,898 controls), large artery stroke (9,219 cases/ 1,503,898 controls), cardioembolic stroke (12,790 cases/ 1,503,898 controls) and small vessel stroke (13,620 cases/ 1,503,898 controls) were retrieved from a multi-ancestry GWAS in the GIGASTROKE consortium.23 Last, we obtained multi-ancestry genetic data for imaging markers of brain health, including brain microbleeds (meta-analysis of 14 GWAS in 3,556 cases and 22,306 controls),24 white matter hyperintensity in 42,310 UKB participants25 and three perivascular space burden traits in 40,095 individuals from the CHARGE consortium.26 All GWAS data used in this study were conducted in multi-ancestry populations, unless otherwise stated.  Catastrophic and any aSAH phenotype generation  To construct binary phenotypes for aSAH and catastrophic aSAH, we used data in the UKB from hospital episode statistics (HES) and death registry (UKB data fields 40001 and 40002) and classified participants as cases based on ICD-9 and ICD-10 codes (UKB data fields 41234 and 41259). Individuals with a hospital admission diagnosis recorded as ICD-9 430 or ICD-10 I60, were classified as aSAH cases. Catastrophic aSAH was defined as fatal aSAH (the codes above recorded as the primary or secondary cause of death) or non-fatal aSAH with at least one of the following diagnoses, defined by ICD-10 codes: hemiparesis (G81), aphasia (R47), apraxia (R48.2), or visual field defects (H53.4), occurring within 7 days after the incident of aSAH. Participants with aSAH occurrence who did not have any complications up to 7 days after aSAH were excluded from the analysis. We also excluded participants with genotype missingness greater than 1.5%, leading to 433 cases/ 476,092 controls for catastrophic aSAH and 1,648 cases/ 476,092 controls for any aSAH with complete data. In analysis of White British ancestry individuals only, there were 350 cases/ 400,610 controls for catastrophic aSAH with complete data. |
|  | e) | Provide details of ethics committee approval and participant informed consent, if relevant | 16 | Ethical approval and participant consent were obtained in the original studies. Patients or the public were not involved in the design, or conduct, or reporting, or dissemination plans of our research. UK Biobank has approval from the North West Multi-centre Research Ethics Committee (MREC) as a Research Tissue Bank (RTB) approval. |
| 5 | **Assumptions** | Explicitly state the three core IV assumptions for the main analysis (relevance, independence and exclusion restriction) as well assumptions for any additional or sensitivity analysis | 5 | To achieve this, MR leverages naturally occurring variation in genetic variants to investigate a causal link between an exposure and an outcome, under three core instrumental variables assumptions: (1) genetic variants used as instruments must be robustly associated with the exposure; (2) genetic variants must not be associated with confounding factors of the exposure-outcome relationship and (3) genetic variants should associate with the outcome exclusively through their effect on the exposure. |
| 6 | **Statistical methods: main analysis** | Describe statistical methods and statistics used |  |  |
|  | a) | Describe how quantitative variables were handled in the analyses (i.e., scale, units, model) | 6 | To construct binary phenotypes for aSAH and catastrophic aSAH, we used data in the UKB from hospital episode statistics (HES) and death registry (UKB data fields 40001 and 40002) and classified participants as cases based on ICD-9 and ICD-10 codes (UKB data fields 41234 and 41259). Individuals with a hospital admission diagnosis recorded as ICD-9 430 or ICD-10 I60, were classified as aSAH cases. Catastrophic aSAH was defined as fatal aSAH (the codes above recorded as the primary or secondary cause of death) or non-fatal aSAH with at least one of the following diagnoses, defined by ICD-10 codes: hemiparesis (G81), aphasia (R47), apraxia (R48.2), or visual field defects (H53.4), occurring within 7 days after the incident of aSAH. Participants with aSAH occurrence who did not have any complications up to 7 days after aSAH were excluded from the analysis. We also excluded participants with genotype missingness greater than 1.5%, leading to 433 cases/ 476,092 controls for catastrophic aSAH and 1,648 cases/ 476,092 controls for any aSAH with complete data. In analysis of White British ancestry individuals only, there were 350 cases/ 400,610 controls for catastrophic aSAH with complete data. |
|  | b) | Describe how genetic variants were handled in the analyses and, if applicable, how their weights were selected | 5 | We selected instrumental single nucleotide polymorphisms (SNPs) that are associated with CSF haptoglobin abundance, below the genome-wide significant level (p < 5 × 10-8) and were located within the HP gene (genomic position chr16:72,088,491-72,094,954, GRCh37/hg19 by Ensembl) or 100kb before the start or after the end of the gene. Genetic variants were clumped using a pair-wise linkage disequilibrium (LD) r2 < 0.1 from the 1000 genomes project phase 3 European LD reference panel.17 If genetic instruments were missing from the outcome datasets, we used proxy SNPs having LD r2 > 0.9 with the missing SNPs. |
|  | c) | Describe the MR estimator (e.g. two-stage least squares, Wald ratio) and related statistics. Detail the included covariates and, in case of two-sample MR, whether the same covariate set was used for adjustment in the two samples | 7 | MR estimates were obtained using the random-effects inverse-variance weighted method 28, which aggregates the effect estimates from the four genetic instrumental variants, weighting them by the inverse of their variance. The MR weighted median29 method was conducted as sensitivity analyses to assess the robustness of the main method findings. To assess potential bias in the MR estimates due to unbalanced horizontal pleiotropy30, we used the p-value of the MR-Egger31 intercept,32 with p-values below 0.05 indicating the presence of pleiotropy. We also calculated Cochran’s Q statistic p-value and the I2 statistic, considering ≤0.05 (Q p-value) and ≥0.75 (I2) indicators of heterogeneity33. |
|  | d) | Explain how missing data were addressed |  |  |
|  | e) | If applicable, indicate how multiple testing was addressed | 7-8 | A p-value of 0.05 was applied as the threshold for statistical significance for primary outcome analysis. For secondary outcomes, we considered a Bonferroni-corrected p-value of 0.05/12 = 0.004 for statistical significance, describing p-values between 0.004 and 0.05 as “nominally significant”. This accounts for the 12 associations tested, including 2 haemorrhagic outcomes, 5 stroke outcomes and 5 brain imaging derived markers (table 1). |
| 7 | **Assessment of assumptions** | Describe any methods or prior knowledge used to assess the assumptions or justify their validity | 7 | . To assess potential bias in the MR estimates due to unbalanced horizontal pleiotropy30, we used the p-value of the MR-Egger31 intercept,32 with p-values below 0.05 indicating the presence of pleiotropy. We also calculated Cochran’s Q statistic p-value and the I2 statistic, considering ≤0.05 (Q p-value) and ≥0.75 (I2) indicators of heterogeneity33. |
| 8 | **Sensitivity analyses and additional analyses** | Describe any sensitivity analyses or additional analyses performed (e.g. comparison of effect estimates from different approaches, independent replication, bias analytic techniques, validation of instruments, simulations) | 8 | Colocalization is a statistical approach to assess whether the genetic predictors of two traits overlap (known as colocalization) or are distinct (known as non-colocalization). A finding of colocalization is consistent with a shared mechanism pathway linking the traits, whereas non-colocalization is indicative of either pleiotropy or linkage disequilibrium with another pathway. The “coloc” method reports several key outputs, including the posterior probability of a causal variant for trait 1 only (PP-H1), the posterior probability of a causal variant for trait 2 only (PP-H2), the posterior probability of non-colocalization (PP-H3), and the posterior probability of colocalization (PP-H4).34 High values (close to 1) of PP-H4 indicate colocalization, which is supportive of a causal relationship; high values of PP-H3 indicate non-colocalization, which opposes a causal relationship; high values of PP-H1 or PP-H2 indicate lack of strong evidence supporting or opposing a causal relationship. In the latter case, the quantity PP-H4/(PP-H3+PP-H4), which represents the posterior probability of colocalization conditional on the presence of a causal variant for both traits, can be calculated to assess whether the evidence favours or opposes colocalization.35 We conducted colocalization analysis to assess whether CSF haptoglobin colocalized with catastrophic aSAH in the HP gene (genomic position chr16:72,088,491-72,094,954, GRCh37/hg19 by Ensembl) or 100kb either way of the gene. |
| 9 | **Software and pre-registration** |  |  |  |
|  | a) | Name statistical software and package(s), including version and settings used | 8 | The R package ‘TwoSampleMR’ v.0.5.7 ^33^ was used to run the analysis. |
|  | b) | State whether the study protocol and details were pre-registered (as well as when and where) |  |  |
|  | **RESULTS** |  |  |  |
| 10 | **Descriptive data** |  |  |  |
|  | a) | Report the numbers of individuals at each stage of included studies and reasons for exclusion. Consider use of a flow diagram | 8 | Catastrophic aSAH was defined as fatal aSAH (the codes above recorded as the primary or secondary cause of death) or non-fatal aSAH with at least one of the following diagnoses, defined by ICD-10 codes: hemiparesis (G81), aphasia (R47), apraxia (R48.2), or visual field defects (H53.4), occurring within 7 days after the incident of aSAH. Participants with aSAH occurrence who did not have any complications up to 7 days after aSAH were excluded from the analysis. We also excluded participants with genotype missingness greater than 1.5%, leading to 433 cases/ 476,092 controls for catastrophic aSAH and 1,648 cases/ 476,092 controls for any aSAH with complete data. In analysis of White British ancestry individuals only, there were 350 cases/ 400,610 controls for catastrophic aSAH with complete data. |
|  | b) | Report summary statistics for phenotypic exposure(s), outcome(s), and other relevant variables (e.g. means, SDs, proportions) | 7-8 | We leveraged summary statistics on CSF haptoglobin relative abundance from a GWAS study in 971 individuals (mean ± standard deviation age of 69.4 ± 9.3 years, 53% women), recruited from the Washington University School of Medicine in St. Louis, United States, including 249 patients with Alzheimer’s disease and 717 cognitively normal controls.18 We obtained genetic data for plasma haptoglobin measured in 35,559 participants of Icelandic ancestry, using the aptamer-based SomaScan v4 platform.19 For the primary outcomes, catastrophic and any aSAH, genetic association estimates were produced using individual level data in the UK Biobank (UKB) under application no. 150618. UKB is a prospective cohort study that involved more than 500,000 participants between the ages of 40 to 69 years.20 Information on the genotyping process, imputation and data quality control has been described elsewhere.21 For any aSAH, we also obtained genetic summary statistics from the largest meta-analysis GWAS to date, conducted in 5,140 aSAH cases and 71,952 controls.22 Genetic association data for intracerebral haemorrhage (cases: 3,391 / controls: 623,600) were obtained from a multi-ancestry GWAS meta-analysis of UK Biobank and FinnGen.23 Genetic data for plasma haemoglobin were also obtained from the same study, measured in 502,921 participants in UK Biobank and Biobank Japan.23 Genetic associations with stroke and stroke subtypes, including all-case stroke (110,182 cases/ 1,503,898 controls), ischemic stroke (86,668 cases/ 1,503,898 controls), large artery stroke (9,219 cases/ 1,503,898 controls), cardioembolic stroke (12,790 cases/ 1,503,898 controls) and small vessel stroke (13,620 cases/ 1,503,898 controls) were retrieved from a multi-ancestry GWAS in the GIGASTROKE consortium.24 Last, we obtained multi-ancestry genetic data for imaging markers of brain health, including brain microbleeds (meta-analysis of 14 GWAS in 3,556 cases and 22,306 controls),25 white matter hyperintensity in 42,310 UKB participants26 and three perivascular space burden traits in 40,095 individuals from the CHARGE consortium.27 All GWAS data used in this study were conducted in multi-ancestry populations, unless otherwise stated. |
|  | c) | If the data sources include meta-analyses of previous studies, provide the assessments of heterogeneity across these studies |  | Not applicable. |
|  | d) | For two-sample MR:  i.  Provide justification of the similarity of the genetic variant-exposure associations between the exposure and outcome samples  ii.  Provide information on the number of individuals who overlap between the exposure and outcome studies | 15 | 1. While our aim was to investigate effects across multi-ancestry populations and specifically within the White British subgroup, the exposure measurements were only available for Europeans, lacking data on multi-ethnic populations. 2. There was no sample overlap in the study. This is evident in the data sources section. |
| 11 | **Main results** |  |  |  |
|  | a) | Report the associations between genetic variant and exposure, and between genetic variant and outcome, preferably on an interpretable scale | 9 | Four SNPs around the HP gene region were used as instruments for CSF haptoglobin (Supplementary table 1). |
|  | b) | Report MR estimates of the relationship between exposure and outcome, and the measures of uncertainty from the MR analysis, on an interpretable scale, such as odds ratio or relative risk per SD difference | 9 | Higher genetically predicted CSF haptoglobin was associated with lower risk of catastrophic aSAH in multi-ancestry (odds ratio [OR]: 0.79, 95% CI: 0.65 to 0.96, p= 0.019) and White British sample analyses (OR: 0.78, 95% CI: 0.63 to 0.95, p= 0.013). There was no association between genetically predicted CSF haptoglobin and the risk of any aSAH, using both UKB participant data (OR: 0.95, 95% CI: 0.84 to 1.07, p= 0.40) and consortium genetic summary statistics (OR: 0.99, 95% CI: 0.86 to 1.13, p= 0.86) for any aSAH. Genetically predicted CSF haptoglobin did not associate (p> 0.05) with stroke or stroke subtypes, white matter hyperintensity, cerebral microbleeds, or other perivascular space burden phenotypes, supporting the specificity of its association with the primary outcome (catastrophic aSAH) (Table 2 and Supplementary table 2). |
|  | c) | If relevant, consider translating estimates of relative risk into absolute risk for a meaningful time period |  |  |
|  | d) | Consider plots to visualize results (e.g. forest plot, scatterplot of associations between genetic variants and outcome versus between genetic variants and exposure) | 19 | Figure 2: Mendelian randomization estimates for the association of higher genetically predicted cerebrospinal fluid (CSF) haptoglobin with catastrophic aneurysmal subarachnoid haemorrhage (aSAH) in multi-ancestry and White British only UK Biobank participants. Mendelian randomization (MR) estimates are expressed as odds ratio per one standard deviation higher genetically predicted CSF haptoglobin. Higher genetically predicted CSF haptoglobin was associated with lower risk of catastrophic aSAH using inverse variance weighted and weighted median MR methods. There was no evidence of pleiotropy or heterogeneity, as indicated by the non-significant Egger intercept and Q-statistic. |
| 12 | **Assessment of assumptions** |  |  |  |
|  | a) | Report the assessment of the validity of the assumptions | 9 | In sensitivity analyses, we observed consistent MR weighted median estimates for catastrophic aSAH (OR: 0.76, 95% CI: 0.60 to 0.97, p= 0.029) and any aSAH (OR: 0.79, 95% CI: 0.63 to 1.00, p= 0.047). |
|  | b) | Report any additional statistics (e.g., assessments of heterogeneity across genetic variants, such as *I^2^*, Q statistic or E-value) | 9 | Cochran’s Q statistic was non-significant (p> 0.05) for all significant associations, indicating no heterogeneity within the effect estimates. Additionally, there was no evidence of horizontal pleiotropy as quantified by the MR-Egger intercept (p> 0.05) (Supplementary table 2). |
| 13 | **Sensitivity analyses and additional analyses** |  |  |  |
|  | a) | Report any sensitivity analyses to assess the robustness of the main results to violations of the assumptions | 9 | In sensitivity analyses, we observed consistent MR weighted median estimates for catastrophic aSAH (OR: 0.76, 95% CI: 0.60 to 0.97, p= 0.029) and any aSAH (OR: 0.79, 95% CI: 0.63 to 1.00, p= 0.047). |
|  | b) | Report results from other sensitivity analyses or additional analyses | 9 | Colocalization analyses did not provide strong evidence for a causal variant for catastrophic aSAH (PP-H1 = 0.99), as variants in the region of interest did not approach necessary significance levels. However, when only considering the hypotheses of colocalization and non-colocalization, the analysis provided suggestive evidence of colocalization (PP-H4/(PP-H3+PP-H4)=0.69) (Supplementary table 3). |
|  | c) | Report any assessment of direction of causal relationship (e.g., bidirectional MR) |  | Not applicable. |
|  | d) | When relevant, report and compare with estimates from non-MR analyses |  | Not applicable. |
|  | e) | Consider additional plots to visualize results (e.g., leave-one-out analyses) |  | Not applicable. |
|  | **DISCUSSION** |  |  |  |
| 14 | **Key results** | Summarize key results with reference to study objectives | 10 | This study investigated the hypothesized relationship between genetically predicted CSF haptoglobin levels and the risk of catastrophic aSAH and related phenotypes. We report that higher genetically predicted CSF haptoglobin levels are associated with lower risk of catastrophic aSAH, but not associated with risk of aSAH, intracerebral haemorrhage, stroke, nor with imaging markers of brain swelling, microbleeds, or white matter injury. There was evidence of colocalization conditional on the presence of a causal variant for both CSF haptoglobin and catastrophic aSAH, which further supported our hypothesis of CSF haptoglobin serving as likely causal risk factor for improving outcomes after aSAH. |
| 15 | **Limitations** | Discuss limitations of the study, taking into account the validity of the IV assumptions, other sources of potential bias, and imprecision. Discuss both direction and magnitude of any potential bias and any efforts to address them | 12 | However, there are important limitations. The use of genetic data limits the direct clinical translatability of our findings as they predominantly capture lifelong effects which do not consider the pharmacokinetic properties of haptoglobin in the CSF in an acute context. Moreover, we instrumented haptoglobin perturbation using aptamer-based protein quantification that do not distinguish between its three proteoforms (Hp 1–1, 2–1, and 2–2)40 but quantifies the relative abundance of (any) haptoglobin. While our aim was to investigate effects across multi-ancestry populations and specifically within the White British subgroup, the exposure measurements were only available for Europeans, lacking data on multi-ethnic populations. Finally, there were no available datasets for CSF haptoglobin in independent comparable populations to replicate our findings. |
| 16 | **Interpretation** |  |  |  |
|  | a) | Meaning: Give a cautious overall interpretation of results in the context of their limitations and in comparison with other studies | 10 | Epidemiological studies on the association between CSF haptoglobin and aSAH risk, post-aSAH complications or related brain imaging phenotypes are limited. The observations from a Japanese single-arm trial in 27 patients that examined the effect of haptoglobin on post-aSAH vasospasm are in concordance with our findings.16 Sixteen (59%) of the 27 patients to whom haptoglobin was administered topically to surrounding intracranial arteries during aneurysmal surgery showed subsequent angiographic evidence of improved vasospasm. However, evidence from this study is weakened by its small sample size, and other important limitations, including lack of a comparator group untreated with haptoglobin and potential selection bias as nine of the 16 responders already showed decreasing vasospasm before haptoglobin treatment. Furthermore, although haptoglobin genotype influences CSF haptoglobin levels36, observational studies have not provided convincing evidence of an association of HP genotype with post-aSAH outcomes10,37. This may be attributed to the physiologically low levels of CSF haptoglobin which may not be sufficient to substantially impact outcomes.10 |
|  | b) | Mechanism: Discuss underlying biological mechanisms that could drive a potential causal relationship between the investigated exposure and the outcome, and whether the gene-environment equivalence assumption is reasonable. Use causal language carefully, clarifying that IV estimates may provide causal effects only under certain assumptions | 11 | Studies in mice have demonstrated reduction in haemoglobin-mediated neurotoxicity and small-vessel vasospasm after haptoglobin treatment.^7,13^ Similarly, a sheep model study suggested that CSF haptoglobin administration halts haemoglobin-induced cerebral vasospasm.^6^ Conversely, following haemoglobin exposure, an in vitro study showed a sevenfold increase in neuronal loss with haptoglobin treatment.^14^ Notably, these neurons expressed the CD163 receptor responsible for the uptake of haptoglobin-haemoglobin complexes,^9^ a function primarily performed in the brain by microglia.^37^ While CD163 expression in human neurons after intracranial haemorrhage is unclear^14^, this has been demonstrated in animal models.^38,39^ Although there are to our knowledge no existing clinical evidence to support this, based on in vitro and animal models neuronal CD163 expression after haemorrhage may give a potential for increased neurotoxicity from haptoglobin-haemoglobin complexes after haptoglobin treatment.^14^ Our findings suggest that higher CSF haptoglobin levels may have a protective effect on downstream outcomes, specifically in reducing the risk of fatality or serious neurological complications once aSAH has already occurred. |
|  | c) | Clinical relevance: Discuss whether the results have clinical or public policy relevance, and to what extent they inform effect sizes of possible interventions | 12 | In conclusion, our results leveraging human genetic data provide further support for a potential effect of higher CSF haptoglobin levels on reducing risk of adverse post-aSAH outcomes. The utility of haptoglobin as a therapeutic is supported by the results from this MR study and well-designed clinical trials are required to further investigate the efficacy and safety of haptoglobin treatment in the context of aSAH management. |
| 17 | **Generalizability** | Discuss the generalizability of the study results (a) to other populations, (b) across other exposure periods/timings, and (c) across other levels of exposure | 12 | While our aim was to investigate effects across multi-ancestry populations and specifically within the White British subgroup, the exposure measurements were only available for Europeans, lacking data on multi-ethnic populations. Finally, there were no available datasets for CSF haptoglobin in independent comparable populations to replicate our findings. |
|  | **OTHER INFORMATION** |  |  |  |
| 18 | **Funding** | Describe sources of funding and the role of funders in the present study and, if applicable, sources of funding for the databases and original study or studies on which the present study is based | 16 | The study was funded by CSL Behring LLC |
| 19 | **Data and data sharing** | Provide the data used to perform all analyses or report where and how the data can be accessed, and reference these sources in the article. Provide the statistical code needed to reproduce the results in the article, or report whether the code is publicly accessible and if so, where | 16 | All genome-wide association study summary data are publicly available in the original studies. UK Biobank individual participant data are available upon appropriate application to the UK Biobank study. |
| 20 | **Conflicts of Interest** | All authors should declare all potential conflicts of interest | 16 | GAK, SA, GK, QA, EF and LT are CSL employees. SA holds stock options at CSL. The rest of the authors declare no competing interests. |

This checklist is copyrighted by the Equator Network under the Creative Commons Attribution 3.0 Unported (CC BY 3.0) license.

1. Skrivankova VW, Richmond RC, Woolf BAR, Yarmolinsky J, Davies NM, Swanson SA, et al. Strengthening the Reporting of Observational Studies in Epidemiology using Mendelian Randomization (STROBE-MR) Statement. JAMA. 2021;under review.

2. Skrivankova VW, Richmond RC, Woolf BAR, Davies NM, Swanson SA, VanderWeele TJ, et al. Strengthening the Reporting of Observational Studies in Epidemiology using Mendelian Randomisation (STROBE-MR): Explanation and Elaboration. BMJ. 2021;375:n2233.
